# Perceived Effectiveness of COVID-19 Preventive Practices and Behavioral Intention: Survey of a Representative Adult Sample in the United States

**DOI:** 10.2196/39919

**Published:** 2023-10-10

**Authors:** Anisah Bagasra, Christopher T Allen, Sara Doan

**Affiliations:** 1 Department of Psychological Science Kennesaw State University Kennesaw, GA United States; 2 Department of Writing, Rhetoric and Cultures Michigan State University East Lansing, MI United States

**Keywords:** health promotion, health communication, health risk behavior, behavioral intention, public health, COVID-19, vaccination, prevention, health education

## Abstract

**Background:**

Using existing models of behavioral health promotion, specifically the Extended Parallel Process Model, previous research has identified factors that may impact engagement in preventive health behaviors during the COVID-19 pandemic such as perceived threat, perceived susceptibility to the threat, perceived severity, and perceived efficacy.

**Objective:**

This study aims to examine the role of perceived effectiveness of COVID-19 preventive behaviors, perceived susceptibility, perceived threat, and perceived severity of COVID-19 in participants’ intentions to engage in Centers for Disease Control (CDC)–recommended individual health behaviors in the first year of the pandemic.

**Methods:**

In October 2020, a representative sample of 506 US adults completed a web-based survey through the RAND American Life Panel.

**Results:**

The study primarily found that participants who perceived that CDC-recommended health practices were effective had stronger intentions to engage in those practices. The second strongest correlate was participants’ perceived severity of COVID-19 across the United States. Perceived effectiveness of recommended practices accounted for the largest variance in behavioral intention. However, analysis of individual behaviors indicated a mismatch in the behaviors perceived to be the most effective (avoiding sick people and mask-wearing) and those participants indicated intention to engage in (throwing away used tissues, avoiding sick people, and coughing into their elbows) in the next 30 days.

**Conclusions:**

The authors recommend tailoring public health messaging to address the perceived threat of COVID-19 and self-efficacy. Thus, health promotion efforts should emphasize the effectiveness of CDC-recommended practices while highlighting the pandemic’s severity. Additionally, rebuilding trust in public health messaging and messengers is necessary to increase perceived self-efficacy. As the COVID-19 pandemic continues, health messaging must continue to promote and build trust in CDC-recommended health practices and educate regarding the efficacy of vaccination and other preventive behaviors.

## Introduction

### Background

Perceptions of both COVID-19 and the effectiveness of recommended health behaviors to prevent the spread of COVID-19 are important factors in reducing personal health risk. The United States’ response to the COVID-19 pandemic contrasts heavily with other countries that engaged in mandated lockdowns and other government-enforced measures [1,2]. In the United States, individual health behavior decisions became the primary method to mitigate the spread of COVID-19 based, in part, on public health education and messaging. Trust in health care practitioners and government institutions has impacted the adoption of health recommendations by the general public [3,4]. Public health messaging’s credibility [5] has been impacted by conflicting and changing messaging from public officials [6,7] and the rapid spread of misinformation about COVID-19 [8,9]. The resulting mortality rates in the United States can be partially attributed to inconsistent adoption and enforcement of public health recommendations [10,11].

Assessing the perceived effectiveness of public health interventions can help experts design and modify health communication strategies to increase engagement in preventive behaviors. Theoretical models of behavioral health, such as the Health Belief Model, the Protective Motivation Theory, and the Extended Parallel Process Model (EPPM), posit perceived efficacy as an important predictor of behavioral engagement [12,13]. Previous studies suggest perceived effectiveness of preventive measures is often mitigated by trust in messaging and messengers, and is key to perceived self-efficacy [12], which plays a significant factor in behavior intention and engagement [13].

To determine what behavioral interventions could lead to wider engagement in preventive health practices, researchers are measuring the perceived efficacy of COVID-19 preventive behaviors [14-17]. Studies measuring the effectiveness of mask-wearing and social distancing indicate the effectiveness of these specific interventions in mitigating community spread [3,18-22]. As researchers continue to study the efficacy of individual protective practices, public health officials rely on community perceptions of effectiveness and community trust in the messages and messengers to persuade individuals into taking action [3,19].

In 2020, the Centers for Disease Control (CDC) recommended preventive health behaviors that formed the foundation for much of the public health messaging communicated within the United States. These recommendations shifted considerably as new information became available to the scientific community regarding COVID-19. For example, in March and April 2020, masking was not initially included in recommended behaviors. As more information became available regarding the transmission of COVID-19, the CDC added masking recommendations to the list of protective behaviors. Further research has examined the effectiveness of different types of masks and facial coverings [20,22] in mitigating the spread of COVID-19. Recommendations regarding protective behaviors, quarantine time periods, testing, and vaccination continue to be updated regularly. Lack of understanding among the public regarding the role of new scientific data contributes to confusion and lack of trust. Shifting messaging regarding protective behaviors also weakens public perceptions of threat, severity, and efficacy, which are key components of models of behavior change.

### Theoretical Framework

One model of behavior change that assesses multiple factors impacting individual health decision-making is the EPPM. The EPPM postulates that perceived threat and efficacy shape individual behaviors to avoid or minimize the perceived threat [23]. Health psychologists and public health officials often use the EPPM as the theoretical foundation when designing health promotion campaigns [24-26]. According to the EPPM, effective health communication messages must credibly communicate the existence of a threat. Conceptually, the EPPM distinguishes between threat as a characteristic of the message (ie, the way in which a threat is communicated in the message) and perceived threat. Threat as a message characteristic refers to features that provide information about the severity of the threat and the target population’s susceptibility; thus, the perceived threat is the subjective evaluation of the threat contained in the message. Perceived threat is a cognitive construct that comprises 2 dimensions: the perceived severity of the threat and one’s perceived susceptibility to the threat. Perceived severity refers to beliefs about the magnitude of the threat and the gravity of its consequences, whereas perceived susceptibility refers to beliefs about the probability of personally experiencing the threat. The model’s second major component is perceived efficacy, which includes both the perceptions of the effectiveness of the behavior and a person’s self-efficacy in their ability to adopt the desired behavior.

According to the EPPM, effective health communication must credibly communicate the existence of a health threat and the efficacy of engaging in the recommended behavior to reduce or eliminate that threat [27]. EPPM has been applied to COVID-19 in a few international studies [25,26,28], suggesting that perceived efficacy is a strong predictor of behavioral engagement [29]. For other highly infectious diseases and respiratory diseases such as influenza and Ebola [23,25-34], the EPPM serves as a lens for understanding the role of threat and efficacy in behavior intention, particularly with a focus on vaccination behavior. Furthermore, communicating threats was less effective in behavior change than convincing individuals of the effectiveness of engaging in health behavior (vaccination) [35-37] particularly for changing behavior around COVID-19 [28,30]. Nazione et al [29] applied the EPPM model to COVID-19 and concluded during the early days of the pandemic that perceived efficacy was the strongest predictor of engaging in preventive behavior. However, few of the recommended behaviors such as mask-wearing were in effect at the time. These studies found relationships between perceived threat, perceived efficacy, and intention to perform certain behaviors such as physical distancing [38]. Most of those studies have been conducted outside the United States with a focus on behavioral intention to engage in social distancing only.

Perceived threat and severity of COVID-19 varies greatly by country of residence, gender, age, sexual orientation, and ethnicity [39-43]. Previous studies suggest that individuals aged 65 or older, women, and minoritized individuals are more likely than others to perceive COVID-19 as a serious personal or communal threat [41]. Masters et al [44] found higher perceived risk among “Millennials” than “Boomers,” but “Boomers” engaged in more social distancing. This suggests that the perceived risk of COVID-19 infection may vary among demographic groups and is not the sole motivating factor in practicing recommended health behaviors. To date, research has consistently shown that people of color are at greater risk of infection, severe illness, and death from COVID-19 than White people; most messaging focused on risks is targeted to older people or those with specific health risks that are exacerbated by structural inequities in wealth, income, and access to health services [45-51].

The success of ongoing public health efforts depends on understanding perceptions of the effectiveness of individual protective behaviors to mitigate the spread of COVID-19. Even as public trust in the government and scientific community has waned, we identify an ongoing need for credible and easy-to-understand public health messaging. Early research on public perceptions of the effectiveness of efforts to reduce the spread of COVID-19 showed general positive perceptions and trust in public health messaging [52]; however, the pandemic’s death toll and infection rate have continued to increase in the United States. Although many preventive health behaviors are no longer enforced by the CDC or the US government, the COVID-19 pandemic continues. The wide availability of misinformation [8,9], and erosion of public health messages’ credibility [5] requires an assessment of public perceptions of COVID-19 to tailor messaging to address beliefs regarding the threat and severity of COVID-19, and the perceived efficacy of individual preventive behaviors.

### Efficacy of CDC-Recommended Behaviors

In October 2020, at the time of this study, the CDC recommended ten behaviors to stop COVID-19’s spread: (1) wash your hands often with soap and water for at least 20 seconds especially after you have been in a public place, or after blowing your nose, coughing, or sneezing; (2) use a hand sanitizer that contains at least 60% alcohol if soap and water are not readily available for hand washing and cover all surfaces of your hands and rub them together until they feel dry; (3) avoid touching your eyes, nose, and mouth with unwashed hands; (4) limit contact with those outside of your household as much as possible; (5) avoid close contact with people who are sick; (6) keep about 6 feet between yourself and others in public settings; (7) cover your mouth and nose with a cloth face cover when around others in public settings; (8) always cover your mouth and nose with a tissue when you cough or sneeze or use the inside of your elbow; (9) throw used tissues in the trash; and (10) clean and disinfect frequently touched surfaces daily. This includes tables, doorknobs, light switches, countertops, handles, desks, phones, keyboards, toilets, faucets, and sinks.

Studies examining the effectiveness of mask-wearing, social distancing, and hand washing globally and within the United States indicate the importance and efficacy of nonpharmaceutical interventions [20,53,54]. Specifically, studies have found that strict lockdown measures lowered fatality rates [21,55,56]. Social distancing encompasses 3 of the CDC recommended behaviors: maintaining 6 feet distance when around other people, avoiding close contact with those outside of one’s household, and avoiding contact with individuals who are sick. The majority of efficacy studies have focused on the effectiveness of social distancing and mask-wearing [20,21,57]. More recent studies have reconfirmed the efficacy of mask-wearing in reducing the risk of COVID-19 infection [57].

Studies of hand hygiene’s efficacy have been sparse but also suggest increased morbidity and mortality among those with lower hand washing adherence in country-level data [48]. Other CDC-recommended behaviors such as sanitizing objects and surfaces, using hand sanitizer when hand washing when water is not available, avoiding touching the eyes, mouth, and nose with unwashed hands, coughing or sneezing into a tissue or elbow, and throwing away used tissues need further examination for efficacy in preventing COVID-19’s spread.

### Data Visualizations and COVID-19 Messaging

Existing messaging about COVID-19 uses visuals to communicate the importance of nonpharmaceutical interventions, visualizing the risk of being infected [58] and the value of social distancing to flatten the curve [59]. This contrasts with messaging from the mainstream media that sometimes downplays transmission rates and ignores issues of race, class, and gender [6]. Much messaging about COVID-19 health behaviors has been designed specifically for social media through visuals [60-63] and to prevent misinformation from spreading [60,64]. Despite the pandemic of misinformation on social media, these platforms remain important for government communications about COVID-19 [63].

Within the messages themselves, COVID-19 is often presented with health gain and loss framing [63], for example, wearing a mask to prevent breathing issues. However, framing around health loss presents ethical issues: overly threatening messages may increase victim-blaming around disability and disease, increasing stigma [65]. Balancing multiple stakeholders’ needs presents a challenge when different demographic groups have varying amounts of trust in scientists’ expertise and values [3]. When persuading disease skeptics, avoid ad hominem attacks and emphasize personal responsibility toward the common good [3]. Connecting the efficacy of preventive health behaviors with self-efficacy creates effective messaging, particularly for social media [66].

For this study, the researchers sought to determine if the perceived effectiveness of CDC practices predicted behavioral intention. The primary research questions posed by the researchers are as follows: (1) can perceived effectiveness be used to predict behavioral intention? (2) What CDC-recommended preventive behaviors do US adults view as most effective in mitigating the spread of COVID-19? The authors hypothesized that after controlling for demographic characteristics: (1) perceiving recommended COVID-19 prevention behaviors as effective would significantly account for intention to engage in those preventive health behaviors and (2) perceiving COVID-19 as a threat and would significantly account for intention to engage in CDC recommended behaviors.

## Methods

### Overview

The COVID-19 Attitudes and Perceptions Survey was fielded via the internet between October 14 and 19, 2020, to participants from RAND’s American Life Panel, a probability-sampled internet-based panel study designed to represent US adults aged 18 and older [67]. RAND’s American Life Panel provides participants with internet access or tools to complete the surveys, allowing members to participate who may normally be excluded from survey research. As such, the RAND American Life Panel made an effective panel to sample from for this study. In total, 506 respondents completed the survey. The sample was comprised of 52% (n=263) women, (mean sample aged 51.4, SD 16.1 years); 77.9% (n=394) White, 20% (n=101) Latinx, 63.9% (n=324) with educational attainment of an Associate degree or less, 47.2% (n=238) with a combined family income of US $59,999 or less during the previous 12 months, an average household size of 2.84 people (SD 1.56), and 97% (n=491) covered by some form of health insurance.

### Ethical Considerations

All study materials and procedures were approved by the Kennesaw State University institutional review board (IRB; approval number: IRB-FY21-13) and the RAND Human Subjects Protection Committee. All participants in this IRB-approved study consented to participation in the survey following procedures for confidential survey participation.

### Measures

#### Perceived Severity of COVID-19

A single survey item was used to assess respondents’ perceived severity of COVID-19 on a scale of 1 (not at all a problem) to 4 (serious problem): “How problematic is COVID-19 in the United States?” (mean 3.61, SD 0.65). Perceived severity of COVID-19 has been measured using various statements referring to the consequences of contracting COVID-19, whether it is life-threatening, and how much of a problem COVID-19 is personally or for your community. As our study was a national study, wording focused on perceptions of COVID-19 as a problem in the United States [68-70]. Participants were therefore asked to rate their perception of the severity of COVID-19 on the national level.

#### Perceived Susceptibility of COVID-19 Infection

A single survey item was used to assess respondents’ perceived susceptibility to COVID-19 infection on a scale of 0-100%: “What do you think is the percent chance that you will get infected with coronavirus in the next month?” (mean 27.09, SD 23.26). Perceived susceptibility therefore measured perceptions of individual risk of contracting COVID-19.

#### Perceived Threat of COVID-19 Infection

A single survey item was used to assess respondents’ perceived threat of a COVID-19 infection on a scale of 1 (not at all concerned) to 5 (extremely concerned): “If you were diagnosed with COVID-19 how concerned would you be about your ability to recover from it?” (mean 3.35, SD 1.28). Recovery from COVID-19 was used as a measure of perceived threat due to misinformation campaigns downplaying the threat of infection as similar to the flu or common cold. Though symptoms can be similar to both the flu and cold the risk of hospitalization, death, longer recovery, and long-term effects (now called post–COVID-19 condition), especially among unvaccinated individuals is higher.

#### Social Desirability

In total, 8 items from the Balanced Inventory of Desirable Responding Short Form [71], Impression Management subscale, were averaged to create a mean score of 5.06 (SD 1.10). We sought to control for socially desirable responses in the study due to the politicization of COVID-19 and associated health promotion behaviors.

#### Perceived Effectiveness of CDC-Recommended COVID-19 Personal Protective Practices

Respondents were asked to rate the perceived effectiveness of 10 CDC-recommended COVID-19 personal protective practices on a scale of 0-100% (“What is the percent chance that this behavior will prevent you from catching COVID-19 over the next month?”). Responses to these items were averaged to create a mean perceived effectiveness score (mean 70.93, SD 22.47), which was included in the analysis as the primary variable of interest.

#### Self-Reported Likelihood of Engaging in CDC-Recommended COVID-19 Personal Protective Practices

Respondents were asked to report their likelihood of engaging in 10 CDC-recommended COVID-19 personal protective practices during the following month, on a scale of 0-100% (“What is the percent chance that you will carry out this behavior over the next month?”). Responses to these items were averaged to create a mean behavioral intention score.

### Data Analysis

A path model was tested using Bayesian estimation in Mplus (version 8.5) software program [72] and following current best practices in Bayesian inference for the use of noninformative priors [73]. Perceived severity of COVID-19, perceived susceptibility of COVID-19 infection, and perceived threat of COVID-19 infection were included in the model as indicators of a latent variable, “Perceived COVID-19 Threat.” Bayesian methods were selected due to their several advantages for both parameter estimation and hypothesis testing relative to frequentist methods [74].

Model fit was assessed holistically using both the posterior predictive *P* value (PPp) and the deviance information criterion. PPp ranges from 0 to 1, with a value of .50 was considered a perfect model fit. PPp values of less than .10, or greater than .90, suggest a poor model fit with data [73].

To test for perceived efficacy as a statistically significant indicator of behavior intention a regression model was estimated using Bayesian estimation in Mplus (version 8.5) software program [72] and following current best practices in Bayesian inference [74]. The following demographic variables were included as covariates in the regression analysis: US census region, Rural (yes/no), gender, age, White (yes/no), Latinx (yes/no), education, family income, household size, and health insurance status (yes/no). To control for potential effects of socially desirable responding, a mean score (mean 5.06, SD 1.10) derived using 8 items from the Impression Management subscale of the Balanced Inventory of Desirable Responding [71] was included as a covariate. The Cronbach α for this scale was .77.

To control for potential effects of respondents’ general perceptions about COVID-19, 3 additional covariates were included in the analysis: perceived COVID-19 severity, perceived COVID-19 susceptibility, and perceived COVID-19 threat. The following survey item was used to assess severity (mean 3.61, SD 0.65) on a scale of 1 (not at all a problem) to 4 (serious problem): “How problematic is COVID-19 in the United States?” The following survey item was used to assess susceptibility (mean 27.09, SD 23.26) on a scale of 0-100%: “What do you think is the percent chance that you will get infected with coronavirus in the next month?”

The survey item used to assess threat perception (mean 3.35, SD 1.28) on a scale of 1 (not at all concerned) to 5 (extremely concerned) was: “If you were diagnosed with COVID-19 how concerned would you be about your ability to recover from it?”

## Results

**Figure 1:**
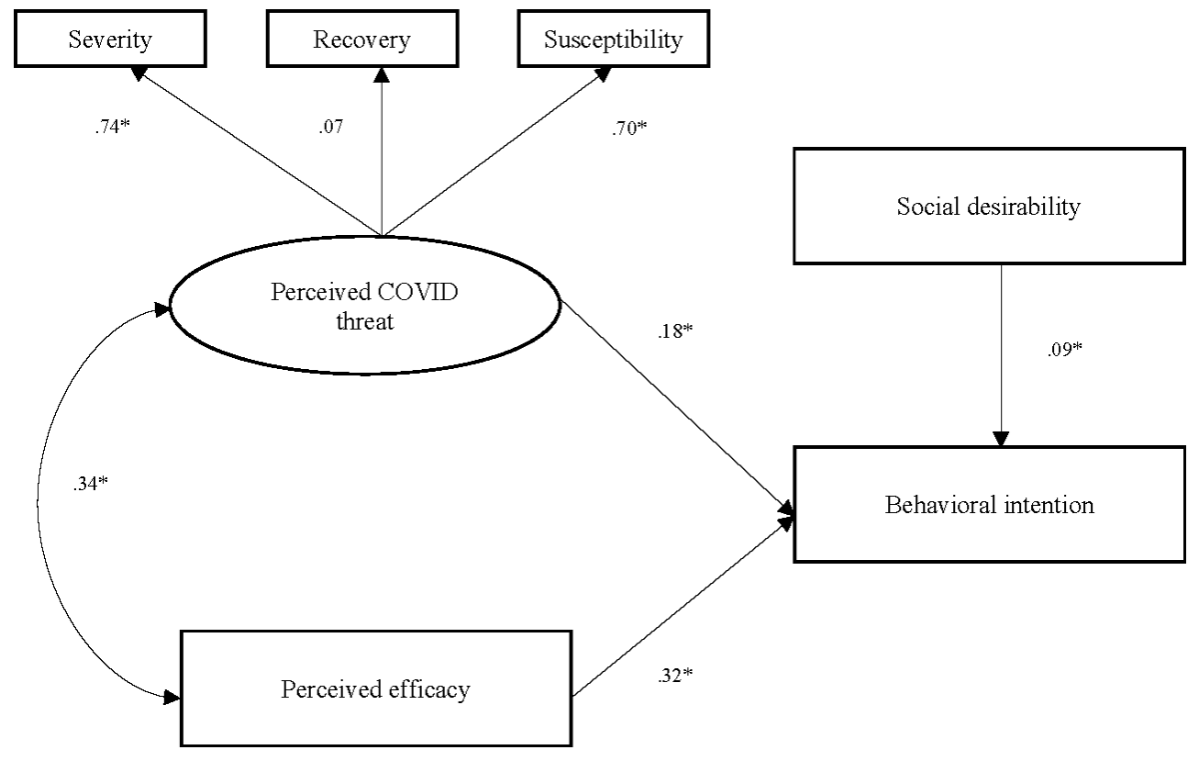
Extended Parallel Process Model (EPPM) path. Note: *=significant at *P*<.01.

The path model demonstrated excellent model fit: PPp=.49, 95% Credibility Interval (–19.65, 22.10); deviance information criterion=1267.92. As predicted by the EPPM, the perceived threat of COVID-19 significantly accounted for participants’ intentions to engage in preventive health practices. However, the perceived efficacy of CDC-recommended preventive health practices was a stronger indicator of intentions to engage in preventive health practices, accounting for 19% of the variance. Path coefficients for each model are displayed in Figure 1.

Results indicate that participants’ perceptions of severity, susceptibility, and infection threat are appropriate indicators of their overall perception of the health threat posed by COVID-19. These results suggest that public health messaging combating COVID-19 misinformation will be effective for calibrating perceptions of the health threat posed by COVID-19. Findings also support previous research, which demonstrated perceived efficacy to be a significant predictor of practicing COVID-19 preventive behavior [21].

The study sample perceived 3 practices as most effective for preventing COVID-19 infection: avoiding close contact with people who are sick (85%), limiting contact with those outside of your household as much as possible (75%), and covering your mouth and nose with a cloth face cover when around others in public settings (74%; See Table 1). These practices diverged from those which the participants reported they would be most likely to engage in during the following month (behavioral intention): throwing used tissues in the trash (93%), avoiding close contact with people who are sick (89%), and always covering your mouth and nose with a tissue when you cough or sneeze or using the inside of your elbow (88%).

After controlling for demographics and socially desirable responding, the Bayesian regression model indicated that US adults’ average perceived effectiveness of CDC-recommended COVID-19 personal protective behaviors was by far the strongest correlate (β=.48; see Table 2) of behavioral intentions. Perceived national COVID-19 severity was the second strongest covariate (β=.19), and perceived personal susceptibility and threat were comparable in strength but negligible (β=.09).

**Table 1:** Perceptions of COVID-19 personal protective practices.

Practice	Perceived effectiveness, mean (SD)	Behavioral intention, mean (SD)
Wash your hands often with soap and water for at least 20 seconds especially after you have been in a public place, or after blowing your nose, coughing, or sneezing.	71.21 (26.48)	81.35 (26.57)
Use a hand sanitizer that contains at least 60% alcohol if soap and water are not readily available for hand washing. Cover all surfaces of your hands and rub them together until they feel dry.	70.41 (26.46)	81.72 (26.23)
Avoid touching your eyes, nose, and mouth with unwashed hands.	71.88 (26.07)	72.00 (28.24)
Limit contact with those outside of your household as much as possible.	75.02 (26.68)	69.62 (30.12)
Avoid close contact with people who are sick.	85.34 (18.94)	88.72 (20.66)
Keep about 6 feet between yourself and others in public settings.	70.17 (26.49)	80.51 (24.34)
Cover your mouth and nose with a cloth face cover when around others in public settings.	74.19 (29.76)	87.78 (23.01)
Always cover your mouth and nose with a tissue when you cough or sneeze or use the inside of your elbow.	63.81 (35.84)	88.04 (21.46)
Throw used tissues in the trash.	61.27 (37.82)	92.72 (17.37)
Clean and disinfect frequently touched surfaces daily. This includes tables, doorknobs, light switches, countertops, handles, desks, phones, keyboards, toilets, faucets, and sinks.	66.10 (30.79)	65.19 (33.90)

**Table 2:** Bayesian regression model results (Standardized): behavioral efficacy.

Indicator	Estimate	Posterior (SD)	*P* (1-tailed)	95% credible interval
US census region	–0.030	0.034	.19	–0.096 to 0.037
Currently live in a rural area (reference=rural)	0.055	0.033	.048	–0.009 to 0.118
Gender (reference=male)	0.082	0.033	.007^a^	0.017 to 0.146
Age	–0.038	0.039	.16	–0.115 to 0.039
Education	0.006	0.037	.43	–0.067 to 0.079
Latinx (reference=not Latinx)	–0.062	0.034	.04	–0.129 to 0.005
White (reference=not White)	0.021	0.035	.27	–0.047 to 0.089
Total family income	0.078	0.038	.02^a^	0.002 to 0.152
Household size	–0.085	0.036	.008^a^	–0.156 to –0.014
Currently has insurance	0.101	0.033	.001^a^	0.036 to 0.166
Impression management	0.208	0.032	<.001^a^	0.144 to 0.270
Perceived COVID-19 severity	0.187	0.038	<.001^a^	0.112 to 0.260
Perceived COVID-19 susceptibility	0.090	0.040	.01^a^	0.012 to 0.167
Perceived COVID-19 threat	0.093	0.035	.004^a^	0.026 to 0.162
Perceived effectiveness	0.480	0.031	<.001^a^	0.418 to 0.539
Model *R*^2^	0.505	0.027	<.001	0.449 to 0.555

^a^Significant at *P*<.03.

## Discussion

### Principal Findings

These survey responses, collected from a nationally representative sample of US adults, indicated that perceived efficacy of COVID-19 prevention behaviors overall correlated with intention to engage in those behaviors. However, the CDC’s recommended practices which respondents perceived to be most effective at preventing COVID-19 infection did not always correspond to their behavioral intention in the next 30 days. Findings suggest that although perceived efficacy is a strong indicator of behavioral intention, the rates of reported behavioral intention for the behaviors perceived to be most effective (social distancing and mask-wearing) were lower than for other CDC-recommended behaviors such as throwing away tissues and covering one’s mouth when coughing or sneezing. The lowest behavioral intention related to social distancing and disinfecting frequently touched surfaces. For ongoing COVID-19 mitigation efforts, especially vaccination strategies, and future public health crises, the authors recommend designing targeted, evidence-based public health messaging to increase trust in public health promotion efforts and willingness to engage in preventive behaviors.

These findings suggest that public health messaging should focus on highlighting the effectiveness of prevention efforts such as social distancing and mask-wearing to persuade people to engage in behaviors they believe to be effective. The behaviors that participants perceive to be most effective in mitigating COVID-19’s spread are also the behaviors most studied for efficacy in the current body of literature [20,21,57]. Despite sound scientific evidence of efficacy for these behaviors, the public has received contradictory information about mask-wearing and social distancing from different sources throughout the pandemic, which may influence behavioral intention. Consistent messaging from credible sources regarding efficacy is important to reduce the mismatch in efficacy and intention identified in this study.

### Implications and Recommendations

To persuade people to engage in the recommended personal protective practices, public health promotion efforts should emphasize the pandemic’s severity throughout the United States. Severity might be emphasized through facts and statistics related to the United States having the highest death toll of all nations, the severity of COVID-19 for certain age groups in the form of mortality or hospitalization rates, or emphasizing the average recovery time for people infected. Furthermore, as rates of COVID-19 infection vary across time and place, health promotion efforts should be tailored to reflect current risk for a given population.

Shifting messaging from fear-based appeals or from overemphasizing personal responsibility to messages of efficacy may also be effective strategies for combating misinformation and encouraging behavioral uptake [75]. Much has been learned during the COVID-19 pandemic about effectively communicating through data visualizations [76]. The authors recommend translating academic findings on efficacy into plain language that can be communicated through infographics and data visualizations that humanize the data and messaging. Although we did not collect data specifically asking participants’ political affiliations, COVID-19 behaviors and communication were heavily politicized by the US government [77]. Across the United States, political party affiliations, education levels, and perceived severity of COVID-19 have been correlated to distrust in government and scientific communication [3,78,79].

The lack of conclusive, available data on the effectiveness of handwashing and sanitizing, and the limited data on mask-wearing and social distancing contributes to an ongoing lack of trust in public health messaging and officials. Data on the success of preventive behaviors should be shared in lay language. Hornik and colleagues [80] similarly conclude that public health campaigns should focus on the effectiveness of health behavior rather than attempt to debunk misinformation.

As vaccination has become the focus of current messaging campaigns, members of the public may be receiving fewer messages regarding COVID-19 as an ongoing threat or the effectiveness of individual behaviors in reducing transmission. Continued utilization of multiple forms of media for health promotion messaging including radio, television, and social media emphasizing both the efficacy of CDC-recommended behaviors and personal efficacy, while reiterating the ongoing threat from COVID-19 infection, is necessary to offset surges during vaccination efforts.

**Figure 2:**
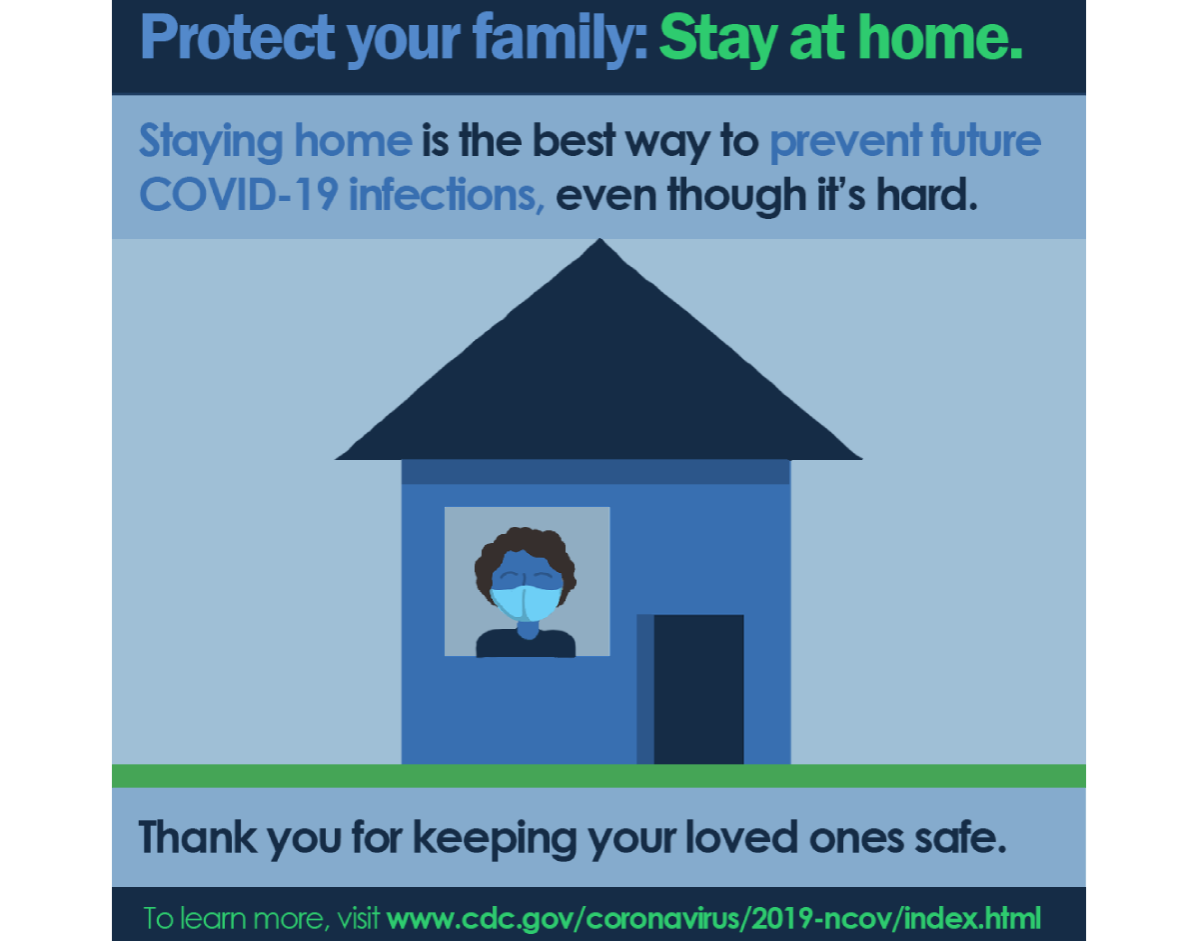
Messaging that acknowledges the effectiveness of preventive health behaviors counters fear-based messaging that undermines public trust. For a different part of this project, the authors created this messaging based on the Extended Parallel Process Model (EPPM). Artist credit (redacted for review).

Misinformation, especially when shared via social media, causes people to underestimate COVID-19’s severity, leading to risky behavior [3,79]. Translating scientific findings into easily digestible visual aids and sound bites may also help to counter misinformation that uses similar methods (Figure 2). During COVID-19, much government messaging about preventive health behaviors has been individualistic, often using fear-based arguments to emphasize the dangers of COVID-19. However, fear-based messaging about chronic illnesses, in general, has been critiqued for emphasizing personal risk and responsibility over larger structural inequities such as race, class, and disability status [80]. Shifting to narratives around the efficacy of preventive health behaviors [65] would begin to alleviate these ethical issues and may be a more effective strategy for communicating about COVID-19, particularly on social media [37].

Based on study findings, the authors recommend that health messaging from trusted sources especially members of the medical community, continue to emphasize the effectiveness of mask-wearing, social distancing from infected persons, COVID-19 testing, getting tested after a known exposure, and hand hygiene as high impact individual behaviors that can be engaged in to reduce the spread of COVID-19. Maintaining consistent, evidence-based messaging as SARS-CoV-2 continues to mutate and cases occasionally spike in communities with lower vaccination rates can increase behavior engagement.

### Study Limitations

This study’s strengths included the use of a nationally representative sample of US adults and the inclusion of survey items that mirrored language used by the CDC (at the time of data collection) to describe COVID-19 prevention practices. Limitations include the small (though representative) sample size, the use of an exclusively web-based survey format, and the availability of the survey solely in English. Though the study was representative, the researchers acknowledge that a larger sample of underrepresented groups who have been disproportionately impacted by COVID-19, especially African Americans and those identifying as Hispanic or Latinx, would have allowed for additional analysis of the impact of demographics on perceived efficacy and behavioral intention. This study also focused on future behavioral intentions rather than behavioral engagement. It should also be noted that, due to third-party survey restrictions, measurement of the latent variable “Perceived COVID Threat” was done using only 3 items. Though model fit statistics indicate this was not empirically tenuous in this study, the authors acknowledge that threat (and its perception) is a multifaceted construct that is typically assessed using a more comprehensive set of items. Additionally, previous research indicates that using percentage scales to assess threat risk—as was done in this study to assess perceived susceptibility—may result in bias as respondents may underestimate a threat rated at the scale’s midpoint [63]. This study relied on survey development in partnership with RAND and limitations on survey length. Therefore, concepts like perceived severity and perceived susceptibility were operationalized and measured using single questions. Perceived severity focused on perceptions of COVID-19 on the national level and perceived susceptibility focused on individual risk. Additionally, the perceived threat item addressed individuals’ concerns regarding recovery from a COVID-19 infection, which differs from operationalizations used in some other studies where threat results from the combination of perceived severity and susceptibility [81]. Similarly, due to survey length limitations, both self-efficacy and response efficacy—2 constructs that are involved in the behavior change process—were excluded from this study. Additional empirical work is needed to replicate the findings of this study with the inclusion of these constructs as they may elucidate important ways that the perceived efficacy of behavioral responses to COVID-19 may be across individuals. Participant responses to these single survey items may have been shaped by their experiences with COVID-19 up until that point, and their observation of the pandemic through news media. Using more than a single item to operationalize perceived severity, threat, and susceptibility would be ideal in future research. Future studies may wish to use an alternative response format in order to validate the findings presented here. Data were collected before the widespread distribution of COVID-19 vaccines; it is unknown how the timing of data collection influenced survey responses. Further research is needed to understand if the perceived efficacy of the CDC-recommended behaviors has shifted over time.

The findings of this study and previous studies suggest the viability of using aspects of the EPPM model to design and implement health promotions. The application of the EPPM model to COVID-19, similar to other infectious diseases can assist health professionals, the government, schools, and businesses in encouraging preventive behaviors. Though current COVID-19 infections tend to be less severe in vaccinated individuals, the medical community is currently preparing for ongoing surges and future mutations that may increase the severity and infectiousness of COVID-19, as well as the possibility of other pandemics. The development of timely and effective models that address cognitive aspects of individual decision-making in the face of health threats is vital to ongoing public health efforts.

## Abbreviations

CDC: Centers for Disease Control
EPPM: Extended Parallel Process Model
IRB: institutional review board
PPp: posterior predictive *P* value

## References

[1] Powell MA, Erwin PC, Bermejo PM (2021). Comparing the COVID-19 responses in Cuba and the United States. Am J Public Health.

[2] Rozanova L, Temerev A, Flahault A (2020). Comparing the scope and efficacy of COVID-19 response strategies in 16 countries: an overview. Int J Environ Res Public Health.

[3] Evans JH, Hargittai E (2020). Who doesn‘t trust fauci? the public‘s belief in the expertise and shared values of scientists in the COVID-19 pandemic. Socius.

[4] Bagasra AB, Doan S, Allen CT (2021). Racial differences in institutional trust and COVID-19 vaccine hesitancy and refusal. BMC Public Health.

[5] Dhanani LY, Franz B (2020). The role of news consumption and trust in public health leadership in shaping COVID-19 knowledge and prejudice. Front Psychol.

[6] Saqr M, Wasson B (2020). COVID-19: lost opportunities and lessons for the future. Int J Health Sci (Qassim).

[7] Nagler RH, Vogel RI, Gollust SE, Rothman AJ, Fowler EF, Yzer MC (2020). Public perceptions of conflicting information surrounding COVID-19: results from a nationally representative survey of U.S. adults. PLoS One.

[8] Doan S (2020). Misrepresenting COVID-19: lying with charts during the second golden age of data design. J Bus Tech Commun.

[9] Kim HK, Ahn J, Atkinson L, Kahlor LA (2020). Effects of COVID-19 misinformation on information seeking, avoidance, and processing: a multicountry comparative study. Sci Commun.

[10] Strand MA, Shyllon O, Hohman A, Jansen RJ, Sidhu S, McDonough S (2022). Evaluating the association of face covering mandates on COVID-19 severity by state. J Prim Care Community Health.

[11] Stoto MA, Schlageter S, Kraemer JD (2022). COVID-19 mortality in the United States: it's been two Americas from the start. PLoS One.

[12] Pförtner TK, Dohle S, Hower KI (2022). Trends in educational disparities in preventive behaviours, risk perception, perceived effectiveness and trust in the first year of the COVID-19 pandemic in Germany. BMC Public Health.

[13] Scholz U, Freund AM (2021). Determinants of protective behaviours during a nationwide lockdown in the wake of the COVID-19 pandemic. Br J Health Psychol.

[14] Glanz K, Rimer BK, Viswanath K (2015). Health Behavior: Theory, Research, and Practice, Fifth Edition.

[15] Nakayachi K, Ozaki T, Shibata Y, Yokoi R (2022). A comparison of perceived effectiveness of preventive behaviors against COVID-19 between the public and medical experts: not so different in means, but in distributions. J Health Psychol.

[16] Serpas DG, Ignacio DA (2022). COVID-19 fear mediates the relationship between perceived risk and preventive behaviors: the moderating role of perceived effectiveness. Psychol Health.

[17] Thompson J, Squiers L, Frasier AM, Bann CM, Bevc CA, MacDonald PDM, McCormack LA (2022). Americans' attitudes toward COVID-19 preventive and mitigation behaviors and implications for public health communication. Am J Health Promot.

[18] Kasting ML, Head KJ, Hartsock JA, Sturm L, Zimet GD (2020). Public perceptions of the effectiveness of recommended non-pharmaceutical intervention behaviors to mitigate the spread of SARS-CoV-2. PLoS One.

[19] Bogart LM, Ojikutu BO, Tyagi K, Klein DJ, Mutchler MG, Dong L, Lawrence SJ, Thomas DR, Kellman S (2021). COVID-19 related medical mistrust, health impacts, and potential vaccine hesitancy among black americans living with HIV. J Acquir Immune Defic Syndr.

[20] Brainard J, Jones NR, Lake IR, Hooper L, Hunter PR (2020). Community use of face masks and similar barriers to prevent respiratory illness such as COVID-19: a rapid scoping review. Euro Surveill.

[21] Matrajt L, Leung T (2020). Evaluating the effectiveness of social distancing interventions to delay or flatten the epidemic curve of coronavirus disease. Emerg Infect Dis.

[22] Hemmer CJ, Hufert F, Siewert S, Reisinger E (2021). Protection from COVID-19–the efficacy of face masks. Dtsch Arztebl Int.

[23] Botta RA, Dunker K, Fenson-Hood K, Maltarich S, McDonald L (2013). Using a relevant threat, EPPM and interpersonal communication to change hand-washing behaviours on campus. J Commun Healthc.

[24] Witte K (1992). Putting the fear back into fear appeals: the extended parallel process model. Commun Monogr.

[25] Roberto AJ (2013). Editor's note for the extended parallel process model: two decades later. Health Commun.

[26] Campo S, Askelson NM, Carter KD, Losch M (2012). Segmenting audiences and tailoring messages. Soc Marketing Q.

[27] Shi JJ, Smith SW (2016). The effects of fear appeal message repetition on perceived threat, perceived efficacy, and behavioral intention in the extended parallel process model. Health Commun.

[28] Lithopoulos A, Liu S, Zhang CQ, Rhodes RE (2021). Predicting physical distancing in the context of COVID-19: a test of the extended parallel process model among Canadian adults. Can Psychol / Psychol Can.

[29] Nazione S, Perrault E, Pace K (2021). Impact of information exposure on perceived risk, efficacy, and preventative behaviors at the beginning of the COVID-19 pandemic in the United States. Health Commun.

[30] Jahangiry L, Bakhtari F, Sohrabi Z, Reihani P, Samei S, Ponnet K, Montazeri A (2020). Risk perception related to COVID-19 among the Iranian general population: an application of the extended parallel process model. BMC Public Health.

[31] Yang J, Wu X, Sasaki K, Yamada Y (2020). Changing health compliance through message repetition based on the extended parallel process model in the COVID-19 pandemic. PeerJ.

[32] Roberto AJ, Mongeau PA, Liu Y, Hashi EC (2019). "Fear the flu, not the flu shot": a test of the extended parallel process model. J Health Commun.

[33] Callis A, Carter VM, Ramakrishnan A, Albert AP, Conteh L, Barrie AA, Fahnbulleh L, Koroma MM, Saidu S, Williams O, Samai M (2018). Lessons learned in clinical trial communication during an Ebola outbreak: the implementation of STRIVE. J Infect Dis.

[34] Figueroa ME (2017). A theory-based socioecological model of communication and behavior for the containment of the Ebola epidemic in Liberia. J Health Commun.

[35] Cameron KA, Rintamaki LS, Kamanda-Kosseh M, Noskin GA, Baker DW, Makoul G (2009). Using theoretical constructs to identify key issues for targeted message design: African American seniors' perceptions about influenza and influenza vaccination. Health Commun.

[36] Prati G, Pietrantoni L, Zani B (2012). Influenza vaccination: the persuasiveness of messages among people aged 65 years and older. Health Commun.

[37] Ort A, Fahr A (2018). Using efficacy cues in persuasive health communication is more effective than employing threats—an experimental study of a vaccination intervention against Ebola. Br J Health Psychol.

[38] Ciancio A, Kämpfen F, Kohler IV, Bennett D, Bruine de Bruin W, Darling J, Kapteyn A, Maurer J, Kohler HP (2020). Know your epidemic, know your response: early perceptions of COVID-19 and self-reported social distancing in the United States. PLoS One.

[39] Helsingen LM, Refsum E, Gjøstein DK, Løberg M, Bretthauer M, Kalager M, Emilsson L, Clinical Effectiveness Research group (2020). The COVID-19 pandemic in Norway and Sweden—threats, trust, and impact on daily life: a comparative survey. BMC Public Health.

[40] Lugo-González IV, Fernández-Vega M, Reynoso-Erazo L, Becerra-Gálvez AL, Pérez-Bautista YY (2020). COVID-19 perception and preventive behaviors: a descriptive, comparative study by severity and perceived risk. Salud Ment.

[41] Niño M, Harris C, Drawve G, Fitzpatrick KM (2021). Race and ethnicity, gender, and age on perceived threats and fear of COVID-19: evidence from two national data sources. SSM Popul Health.

[42] Potter EC, Tate DP, Patterson CJ (2021). Perceived threat of COVID-19 among sexual minority and heterosexual women. Psychol Sex Orientat Gend Divers.

[43] DeSalvo N, Lacasse K, Jackson TE (2022). Gender norms shape perceived threat to self and others and mask wearing behavior in response to COVID-19. Transl Issues Psychol Sci.

[44] Masters NB, Shih SF, Bukoff A, Akel KB, Kobayashi LC, Miller AL, Harapan H, Lu Y, Wagner AL (2020). Social distancing in response to the novel coronavirus (COVID-19) in the United States. PLoS One.

[45] Stokes EK, Zambrano LD, Anderson KN, Marder EP, Raz KM, El Burai Felix S, Tie Y, Fullerton KE (2020). Coronavirus disease 2019 case surveillance—United States, January 22-May 30, 2020. MMWR Morb Mortal Wkly Rep.

[46] Killerby ME, Link-Gelles R, Haight SC, Schrodt CA, England L, Gomes DJ, Shamout M, Pettrone K, O'Laughlin K, Kimball A, Blau EF, Burnett E, Ladva CN, Szablewski CM, Tobin-D'Angelo M, Oosmanally N, Drenzek C, Murphy DJ, Blum JM, Hollberg J, Lefkove B, Brown FW, Shimabukuro T, Midgley CM, Tate JE, CDC COVID-19 Response Clinical Team (2020). Characteristics associated with hospitalization among patients with COVID-19—Metropolitan Atlanta, Georgia, March-April 2020. MMWR Morb Mortal Wkly Rep.

[47] Gold JAW, Wong KK, Szablewski CM, Patel PR, Rossow J, da Silva J, Natarajan P, Morris SB, Fanfair RN, Rogers-Brown J, Bruce BB, Browning SD, Hernandez-Romieu AC, Furukawa NW, Kang M, Evans ME, Oosmanally N, Tobin-D'Angelo M, Drenzek C, Murphy DJ, Hollberg J, Blum JM, Jansen R, Wright DW, Sewell WM, Owens JD, Lefkove B, Brown FW, Burton DC, Uyeki TM, Bialek SR, Jackson BR (2020). Characteristics and clinical outcomes of adult patients hospitalized with COVID-19—Georgia, March 2020. MMWR Morb Mortal Wkly Rep.

[48] Price-Haywood EG, Burton J, Fort D, Seoane L (2020). Hospitalization and mortality among black patients and white patients with Covid-19. N Engl J Med.

[49] Millett GA, Jones AT, Benkeser D, Baral S, Mercer L, Beyrer C, Honermann B, Lankiewicz E, Mena L, Crowley JS, Sherwood J, Sullivan PS (2020). Assessing differential impacts of COVID-19 on black communities. Ann Epidemiol.

[50] Loeb TB, Ebor MT, Smith-Clapham AM, Chin D, Novacek DM, Hampton-Anderson JN, Norwood-Scott E, Hamilton AB, Brown AF, Wyatt GE (2021). How mental health professionals can address disparities in the context of the COVID-19 pandemic. Traumatology (Tallahass Fla).

[51] Raifman MA, Raifman JR (2020). Disparities in the population at risk of severe illness from COVID-19 by race/ethnicity and income. Am J Prev Med.

[52] Seale H, Heywood AE, Leask J, Sheel M, Thomas S, Durrheim DN, Bolsewicz K, Kaur R (2020). COVID-19 is rapidly changing: examining public perceptions and behaviors in response to this evolving pandemic. PLoS One.

[53] Dehning J, Zierenberg J, Spitzner FP, Wibral M, Neto JP, Wilczek M, Priesemann V (2020). Inferring change points in the spread of COVID-19 reveals the effectiveness of interventions. Science.

[54] Szczuka Z, Abraham C, Baban A, Brooks S, Cipolletta S, Danso E, Dombrowski SU, Gan Y, Gaspar T, de Matos MG, Griva K, Jongenelis M, Keller J, Knoll N, Ma J, Miah MAA, Morgan K, Peraud W, Quintard B, Shah V, Schenkel K, Scholz U, Schwarzer R, Siwa M, Szymanski K, Taut D, Tomaino SCM, Vilchinsky N, Wolf H, Luszczynska A (2021). The trajectory of COVID-19 pandemic and handwashing adherence: findings from 14 countries. BMC Public Health.

[55] Pachetti M, Marini B, Giudici F, Benedetti F, Angeletti S, Ciccozzi M, Masciovecchio C, Ippodrino R, Zella D (2020). Impact of lockdown on Covid-19 case fatality rate and viral mutations spread in 7 countries in Europe and North America. J Transl Med.

[56] Feyman Y, Bor J, Raifman J, Griffith KN (2020). Effectiveness of COVID-19 shelter-in-place orders varied by state. PLoS One.

[57] Candevir A, Üngör C, Şenel FÇ, Taşova Y (2021). How efficient are facial masks against COVID-19? evaluating the mask use of various communities one year into the pandemic. Turk J Med Sci.

[58] Verhulsdonck G, Shah V (2020). Lean data visualization: considering actionable metrics for technical communication. J Bus Tech Commun.

[59] Amidon TR, Nielsen AC, Pflugfelder EH, Richards DP, Stephens SH (2020). Visual risk literacy in “flatten the curve” COVID-19 visualizations. J Bus Tech Commun.

[60] Hope L (2020). Protecting pandemic conversations: tracing Twitter’s evolving content policies during COVID-19. J Bus Tech Commun.

[61] Kouzy R, Abi Jaoude J, Kraitem A, El Alam MB, Karam B, Adib E, Zarka J, Traboulsi C, Akl EW, Baddour K (2020). Coronavirus goes viral: quantifying the COVID-19 misinformation epidemic on Twitter. Cureus.

[62] Walwema J (2020). The WHO health alert: communicating a global pandemic with WhatsApp. J Bus Tech Commun.

[63] Sleigh J, Amann J, Schneider M, Vayena E (2021). Qualitative analysis of visual risk communication on twitter during the Covid-19 pandemic. BMC Public Health.

[64] Pennycook G, McPhetres J, Zhang Y, Lu JG, Rand DG (2020). Fighting COVID-19 misinformation on social media: experimental evidence for a scalable accuracy-nudge intervention. Psychol Sci.

[65] Guttman N, Salmon CT (2004). Guilt, fear, stigma and knowledge gaps: ethical issues in public health communication interventions. Bioethics.

[66] Gallagher J, Lawrence HY (2020). Rhetorical appeals and tactics in New York Times comments about vaccines: qualitative analysis. J Med Internet Res.

[67] About the Panel. RAND Corporation.

[68] Roccato M, Pacilli MG, Orlando G, Russo S (2022). Masculinity, perceived vulnerability to COVID-19, and adoption of protective behaviors. Sex Cult.

[69] Sobaih AEE, Moustafa F (2022). Panic food purchasing amid COVID-19 pandemic: does the impact of perceived severity, anxiety and self-isolation really matter?. Int J Environ Res Public Health.

[70] Yang D, Wagner AL, Gorin SS (2022). Perceived severity of COVID-19 in a longitudinal study in Detroit, Michigan. Ethn Dis.

[71] Hart CM, Ritchie TD, Hepper EG, Gebauer JE (2015). The Balanced Inventory of Desirable Responding short form (BIDR-16). SAGE Open.

[72] Muthén LK, Muthén B (2017). Mplus User's Guide. Eighth Edition.

[73] Depaoli S, van de Schoot R (2017). Improving transparency and replication in bayesian statistics: the WAMBS-checklist. Psychol Methods.

[74] Wagenmakers EJ, Marsman M, Jamil T, Ly A, Verhagen J, Love J, Selker R, Gronau QF, Šmíra M, Epskamp S, Matzke D, Rouder JN, Morey RD (2018). Bayesian inference for psychology. part I: theoretical advantages and practical ramifications. Psychon Bull Rev.

[75] Muthén B, Asparouhov T (2012). Bayesian structural equation modeling: a more flexible representation of substantive theory. Psychol Methods.

[76] Rosenbaum L (2020). Tribal truce—how can we bridge the partisan divide and conquer Covid?. N Engl J Med.

[77] Jones CL, Jensen JD, Scherr CL, Brown NR, Christy K, Weaver J (2015). The health belief model as an explanatory framework in communication research: exploring parallel, serial, and moderated mediation. Health Commun.

[78] Bishop T, Capan E, Larsen B, Preston R, Sparby EM (2021). Tactical risk communication: observations from teaching and learning about crisis communication during COVID-19. Tech Commun Q.

[79] Latkin CA, Dayton L, Strickland JC, Colon B, Rimal R, Boodram B (2021). An assessment of the rapid decline of trust in US sources of public information about COVID-19. J Health Commun.

[80] Hornik R, Kikut A, Jesch E, Woko C, Siegel L, Kim K Association of COVID-19 misinformation with face mask wearing and social distancing in a nationally representative US sample. ArXiv..

[81] Vacondio M, Priolo G, Dickert S, Bonini N (2021). Worry, perceived threat and media communication as predictors of self-protective behaviors during the COVID-19 outbreak in Europe. Front Psychol.

